# A comparison of survival models for prediction of eight-year revision risk following total knee and hip arthroplasty

**DOI:** 10.1186/s12874-022-01644-3

**Published:** 2022-06-06

**Authors:** Alana R. Cuthbert, Lynne C. Giles, Gary Glonek, Lisa M. Kalisch Ellett, Nicole L. Pratt

**Affiliations:** 1 Quality Use of Medicines and Pharmacy Research Centre, Clinical and Health Sciences, University of South Australia, PO Box 11060, Adelaide, SA 5001 Australia; 2 South Australian Health and Medical Research Institute, Adelaide, SA 5000 Australia; 3 School of Public Health, The University of Adelaide, Adelaide, SA 5005 Australia; 4 School of Mathematical Sciences, The University of Adelaide, Adelaide, SA 5005 Australia

**Keywords:** Prediction model, Machine learning, Time-to-event data, Flexible parametric survival model, Parametric survival model, Random survival forest, Hip replacement, Knee replacement

## Abstract

**Background:**

There is increasing interest in the development and use of clinical prediction models, but a lack of evidence-supported guidance on the merits of different modelling approaches. This is especially true for time-to-event outcomes, where limited studies have compared the vast number of modelling approaches available. This study compares prediction accuracy and variable importance measures for four modelling approaches in prediction of time-to-revision surgery following total knee arthroplasty (TKA) and total hip arthroplasty (THA).

**Methods:**

The study included 321,945 TKA and 151,113 THA procedures performed between 1 January 2003 and 31 December 2017. Accuracy of the Cox model, Weibull parametric model, flexible parametric model, and random survival forest were compared, with patient age, sex, comorbidities, and prosthesis characteristics considered as predictors. Prediction accuracy was assessed using the Index of Prediction Accuracy (IPA), c-index, and smoothed calibration curves. Variable importance rankings from the Cox model and random survival forest were also compared.

**Results:**

Overall, the Cox and flexible parametric survival models performed best for prediction of both TKA (integrated IPA 0.056 (95% CI [0.054, 0.057]) compared to 0.054 (95% CI [0.053, 0.056]) for the Weibull parametric model), and THA revision. (0.029 95% CI [0.027, 0.030] compared to 0.027 (95% CI [0.025, 0.028]) for the random survival forest). The c-index showed broadly similar discrimination between all modelling approaches. Models were generally well calibrated, but random survival forest underfitted the predicted risk of TKA revision compared to regression approaches. The most important predictors of revision were similar in the Cox model and random survival forest for TKA (age, opioid use, and patella resurfacing) and THA (femoral cement, depression, and opioid use).

**Conclusion:**

The Cox and flexible parametric models had superior overall performance, although all approaches performed similarly. Notably, this study showed no benefit of a tuned random survival forest over regression models in this setting.

**Supplementary Information:**

The online version contains supplementary material available at 10.1186/s12874-022-01644-3.

## Background

There is increasing interest in the development and use of clinical prediction models [1]. Accurate prediction models can assist in informed decision-making by estimating a patient’s risk of a health outcome based on their individual characteristics, rather than relying on crude population-level estimates. However, developing an accurate prediction model requires the researcher to choose from many available modelling approaches and limited studies have compared the advantages and disadvantages of each method.

For time-to-event outcomes the Cox model is the most common approach, but parametric survival models, including flexible parametric models, may be preferable depending on the complexity of the data. Alternatively, machine learning methods hold promise of improved prediction accuracy through automatic modelling of non-linearities, interactions, and time-varying effects in predictor variables. These methods make fewer (or no) assumptions about the underlying structure of the data [2], but as a consequence they are generally less efficient and require much larger sample sizes to obtain stable predictions [3]. Another drawback of machine learning methods is lack of interpretability; understanding which variables are important for prediction and how they influence the outcome are critical to the utility of such models [4]. Many machine learning methods provide measures of how ‘important’ each variable is, but do not indicate effect size or direction. A raft of machine learning methods to assist in the development of prediction models are now available, but few studies have systematically compared their performance to traditional regression approaches [5].

An important clinical area for the development of prediction models is joint replacement surgery. Arthroplasty of the hip or knee is an effective treatment for end stage osteoarthritis, an increasingly common disease and one of the leading causes of global disability [6]. While joint replacements are expected to last at least 25 years on average, a small proportion will fail within a shorter time frame and require revision surgery [7, 8]. Revision surgery is defined as the addition, removal or exchange of one or more prosthetic components, and is an unambiguous indication that there are problems with the joint severe enough to require further surgery [9, 10]. Premature revision surgery is a major burden for both patients and the healthcare system, resulting in worse outcomes for patients and billions in hospital costs [11, 12]. Improved prediction of the risk of revision, by taking into account patient-, surgeon- and prosthesis-related factors, will better inform patients of their likely risks when undergoing elective surgery, as well as enable hospitals to predict expected health care burden. Prospective joint replacement recipients are concerned with both their risk of revision surgery and the ways in which their personal characteristics influence this risk, highlighting the need for prediction models that are both accurate and interpretable [13].

In the present study, four survival modelling approaches for predicting time-to-revision within 8 years of joint arthroplasty surgery are compared: Cox regression, parametric regression with a Weibull distribution, flexible parametric regression, and random survival forests. Variable importance rankings from the Cox model and random survival forests are also compared.

## Methods

### Data source

This study used data from elective primary Total Knee Arthroplasty (TKA) and Total conventional Hip Arthroplasty (THA) procedures recorded in the Australian Orthopaedic Association National Joint Replacement Registry (AOANJRR) between 1 July 2003 and 31 December 2017. The Registry collects data on patient age, sex, indication for surgery, and prosthesis type and features, recorded by hospital staff at the time of surgery. Patient comorbidities were identified through record linkage with the Pharmaceutical Benefits Scheme administrative claims database. This database is maintained by the Australian Government Department of Human Services and contains information on the dispensing of prescription medicines. Using probabilistic data linkage, 95% of procedures in the AOANJRR were linked to Pharmaceutical Benefits Scheme data. A total of 47 morbid conditions were identified using the validated Rx-Risk coding of patient prescriptions [14]. A patient was considered to have a morbid condition if they were dispensed at least one medicine indicative of that condition in the 12 months prior to their joint replacement surgery.

The AOANJRR captured approximately 98% of knee and hip arthroplasties in Australia over the study period, including both primary and revision procedures. Revision procedures were identified by internally matching primary and revision procedures on patient information and side of joint replacement (left or right). Revision procedures unable to be matched or performed in another country were not captured. Patient death was identified through record linkage with the National Death Index.

### Participants and variables

The inclusion criteria for TKAs were: primary indication of osteoarthritis, patients aged 45 to 89 years at the time of surgery; minimally and posterior-stabilised prostheses only, and no missing prosthesis attributes.

The inclusion criteria for THAs were: primary indication of osteoarthritis, patients aged 40 to 89 years at the time of surgery; modern bearings (metal-on-cross-linked polyethylene, ceramic-on-cross-linked polyethylene, and ceramic-on-ceramic), and no missing prosthesis attributes.

In addition, only patients receiving concessional benefits were included. These patients are eligible healthcare cardholders or pensioners who pay a lower co-payment towards the cost of medicines subsidised by the Australian Government, and represented 80% of the total joint replacement population. Flowcharts showing details of inclusion criteria are provided in Additional file 1.

Once selection criteria were applied, 321,945 TKA and 151,113 THA procedures (performed in 254,886 and 131,386 patients, respectively) were available for model development and validation. A summary of the study population is given in Table 1 for TKAs and Table 2 for THAs.

**Table 1:** Demographics, comorbid conditions and prosthesis use in all patients undergoing TKA and for those revised within 8 years of surgery

Variable	All TKA *N* = 321,945	Revised *N* = 9819
Median (IQR) age in years	72 (67-78)	70 (64-75)
N (%) female	195,668 (60.8)	5659 (57.6)
N (%) cemented femoral	188,734 (58.6)	5438 (55.4)
N (%) cemented tibial	259,187 (80.5)	7344 (74.8)
N (%) patella used	164,333 (51)	3916 (39.9)
N (%) fixed bearing	257,008 (79.8)	7202 (73.3)
N (%) minimally stabilised	234,827 (72.9)	6669 (67.9)
N (%) cross-linked polyethylene	107,456 (33.4)	2080 (21.2)
N (%) computer navigated	60,164 (18.7)	1551 (15.8)
N (%) anticoagulants	42,398 (13.2)	1281 (13)
N (%) antiplatelet medications	64,011 (19.9)	2091 (21.3)
N (%) anxiety	34,863 (10.8)	1462 (14.9)
N (%) Arrhythmia	18,373 (5.7)	568 (5.8)
N (%) congestive heart failure	25,281 (7.9)	833 (8.5)
N (%) depression	83,445 (25.9)	3165 (32.2)
N (%) diabetes	47,041 (14.6)	1442 (14.7)
N (%) Gastro-oesophageal reflux disease	156,820 (48.7)	5058 (51.5)
N (%) glaucoma	20,382 (6.3)	527 (5.4)
N (%) gout	30,540 (9.5)	997 (10.2)
N (%) hyperlipidaemia	160,478 (49.8)	4817 (49.1)
N (%) hypertension	181,127 (56.3)	5325 (54.2)
N (%) hypothyroidism	33,546 (10.4)	1053 (10.7)
N (%) ischaemic heart disease (angina)	21,925 (6.8)	826 (8.4)
N (%) ischaemic heart disease (hypertension)	117,801 (36.6)	3399 (34.6)
N (%) osteoporosis/Paget’s	30,706 (9.5)	844 (8.6)
N (%) pain	151,805 (47.2)	5514 (56.2)
N (%) inflammation pain	176,883 (54.9)	5949 (60.6)
N (%) chronic airways disease	71,122 (22.1)	2373 (24.2)
N (%) steroid responsive	54,271 (16.9)	1906 (19.4)

**Table 2:** Demographics, comorbid conditions and prosthesis use in all patients undergoing THA and for those revised within 8 years of surgery

Variable	All THA *N* = 151,113	Revised *N* = 4415
Median (IQR) age in years	73 (68-79)	73 (67-78)
N (%) female	90,153 (59.7)	2498 (56.6)
N (%) cemented femoral	66,537 (44)	1534 (34.7)
N (%) bearing surface		
Ceramic/ceramic	28,886 (19.1)	1036 (23.5)
Ceramic/cross-linked polyethylene	27,249 (18)	698 (15.8)
Metal/cross-linked polyethylene	94,978 (62.9)	2681 (60.7)
N (%) head size		
≤ 28 mm	29,724 (19.7)	995 (22.5)
32 mm	64,854 (42.9)	1761 (39.9)
36 mm	53,376 (35.3)	1558 (35.3)
≥ 40 mm	3159 (2.1)	101 (2.3)
N (%) anticoagulants	20,990 (13.9)	658 (14.9)
N (%) antiplatelet medications	27,645 (18.3)	899 (20.4)
N (%) anxiety	16,146 (10.7)	627 (14.2)
N (%) arrhythmia	9092 (6)	290 (6.6)
N (%) congestive heart failure	11,708 (7.7)	410 (9.3)
N (%) depression	37,159 (24.6)	1402 (31.8)
N (%) diabetes	16,689 (11)	494 (11.2)
N (%) Gastro-oesophageal reflux disease	65,911 (43.6)	2194 (49.7)
N (%) glaucoma	9824 (6.5)	286 (6.5)
N (%) gout	11,325 (7.5)	372 (8.4)
N (%) hyperlipidaemia	70,365 (46.6)	1990 (45.1)
N (%) hypertension	78,101 (51.7)	2270 (51.4)
N (%) hypothyroidism	14,282 (9.5)	430 (9.7)
N (%) ischaemic heart disease (angina)	9576 (6.3)	309 (7)
N (%) ischaemic heart disease (hypertension)	50,495 (33.4)	1512 (34.2)
N (%) osteoporosis/Paget’s	15,157 (10)	501 (11.3)
N (%) pain	82,787 (54.8)	2714 (61.5)
N (%) inflammation pain	83,078 (55)	2714 (61.5)
N (%) chronic airways disease	29,633 (19.6)	967 (21.9)
N (%) steroid responsive	22,730 (15)	805 (18.2)

### Modelling approaches

The semi-parametric Cox proportional hazards model is the most widely used model for predicting time-to-event outcomes. When fitting a Cox model the distributional form of the baseline hazard does not need to be specified, an advantageous feature when the hazard function is unknown or complex. However, as the baseline hazard is unspecified, the Cox model cannot directly provide an estimate of the survival function. In order to predict survival probabilities, an estimate of the baseline cumulative hazard needs to be calculated (using the Nelson-Aalen estimator or similar) and combined with the coefficients from the Cox model. The time points for which the Cox model can predict outcomes are then restricted to the discrete time points at which events occurred in the data used to develop the model. This means it is not possible to extrapolate predictions from the Cox model, or make predictions precisely at time points where no events occurred.

Unlike the Cox model, which makes no assumptions about the distributional form of the hazard function, parametric survival models assume the baseline hazard follows a particular distribution, such as the Weibull, logistic, log-logistic or log-normal. The Weibull distribution on a proportional hazards scale is commonly chosen for use in health data. An advantage of parametric models is that they can be specified fully by a mathematical equation. Hence predictions with parametric models can be made directly using this equation. This means it is possible to make predictions for any time point, and even extrapolate survival predictions, rather than being restricted to the discrete time points at which events occurred. However, if the shape of the baseline hazard function is unknown or complex, then it can be challenging to find a distribution that adequately describes it, potentially resulting in less accurate predictions. The Royston-Parmar flexible parametric model was introduced to overcome the limitations of parametric survival models by allowing flexible modelling of the baseline hazard [15]. Rather than modelling the baseline hazard with a pre-specified distribution, a restricted cubic spline is used to flexibly model the baseline log cumulative hazard function. This can be thought of as a hybrid of the Cox and parametric survival models, as the model can be specified mathematically without imposing the restrictions of a particular distribution on the hazard function. The complexity of the function used to model the baseline hazard is determined by the number of knots in the restricted cubic spline. It has been shown that the model is relatively robust to the number and placement of knots; more than 5 knots are rarely required, but up to 7-8 knots may be required for complex variables such as time [16, 17].

Despite the benefits of flexible parametric models, they are rarely used for prognostic models in medical settings and have not been systematically compared to other survival analysis approaches [18].

Both the Cox and parametric models on the proportional hazards scale assume that the effect of covariates is constant over time, which is rarely a realistic assumption in medical settings. Parametric and semi-parametric approaches also require interactions between variables to be explicitly specified, which may be intractable when the number of predictors is large. In contrast, the random survival forest algorithm is a fully non-parametric machine learning approach that does not assume proportional hazards and can automatically account for possible interaction effects [19]. Introduced in 2008, it is an extension of the random forest algorithm that makes predictions for new patients by aggregating predicted survival curves from a series of survival trees. Random survival forests can also provide fully nonparametric measures of variable importance [20].

### Statistical analysis

The outcome of interest was time-to-first revision within 8 years of primary joint arthroplasty. Patient age, sex, prosthesis characteristics, and comorbidities with at least 5% prevalence in the analysis dataset were used in the model, resulting in 29 variables for TKA revision and 26 for THA revision (Tables 1 and 2). In the Cox and parametric models, age was treated as a continuous variable and modelled with a restricted cubic spline with four knots. All other variables included in the prediction models were categorical. Patients were censored at the time of database closure (31 December 2017) or death. Patient death was treated as a censoring event as ignoring competing risks has been shown to have a negligible impact on prediction modelling of time-to-revision following joint replacement surgery [21]. All analyses assumed censoring was independent of event rates, conditional on covariates included in the model. If a patient had bilateral TKAs or THAs, each side was treated as a separate unilateral procedure, which has been shown to have a negligible effect on model estimates [22]. Seven knots were used for modelling the log cumulative hazard in the flexible parametric model, with knots placed at the default location of equally spaced quantiles of the log uncensored survival times. The crude cumulative incidence of revision was calculated using the Aalen-Johansen estimator with patient death treated as a competing event [23]. The baseline hazard was estimated using the bshazard function in R, which calculates smoothed, non-parametric estimate of hazard function using B-splines [24].

Random survival forests were grown using log-rank splitting with 300 trees. Two parameters were tuned: the terminal node size and the number of variables considered for splits when growing the survival trees. Full details of the tuning process are provided in Additional file 3.

### Model performance

The eight-year prediction performance of the four modelling approaches was averaged across 10 repetitions of 10-fold cross-validation. Cross-validation was used to reduce the bias and variability of estimated performance that may result from using a single testing/training split and ensure the results obtained did not depend arbitrarily on the random split of the data chosen [25, 26]. 95% confidence intervals for performance metrics were calculated by computing a standard normalised interval around the mean using the different values estimated within each fold. Normality was assessed using quantile-quantile plots and found to be a reasonable assumption for all performance metrics.

Model discrimination was assessed using Harrell’s concordance index (c-index). The c-index estimates the probability that, for a randomly selected pair of patients, the patient with highest predicted risk fails first. The value of the c-index ranges from 0.5 to 1, with a value of 1 implying perfect discrimination and 0.5 representing a model that is no better than random guessing.

Calibration was assessed using smoothed calibration curves to compare the proportion of observed and predicted events at 8 years [27]. A calibration curve that closely follows the 45-degree identity line indicates a good match between predicted and observed values. The smoothed plots were generated using a Cox model with predicted probabilities modelled using a restricted cubic spline with four knots. Calibration was assessed for predicted probabilities ranging from the first percentile to the 99th percentile. Calibration curves were calculated using the same grid of predicted probabilities for each fold and repetition of cross-validation and then averaged across 10 repetitions of 10-fold cross-validation. A numeric summary of the calibration curve, the Integrated Calibration Index (ICI), was also calculated, with lower values indicating a smaller average difference between the observed and predicted probabilities [28]. Additional calibration plots comparing the average predicted survival curves from each method to the Kaplan-Meier curve are presented in Additional file 4.

The overall performance of each model was assessed using the Index of Prediction Accuracy (IPA), derived as 1-(model Brier score/null model Brier score), where the null model is the Kaplan-Meier estimator [29]. The Brier score measures the average squared distance between the observed event status and predicted event probability for each individual at a single point in time, thereby providing a combined measure of discrimination and calibration. Higher IPA values imply better model fit, with 100% representing a perfect model. Values ≤0 indicate the model performs no better than the population-level estimate. The IPA was integrated over 8 years to summarise model performance in a single numeric value, as well as calculated at several time points and presented graphically to show the predictive performance of the modelling approaches over the eight-year period.

The c-index and IPA were weighted using inverse probability of censoring to correct for bias introduced by censoring [30–32]. All performance metrics were estimated using ten repetitions of 10-fold cross-validation.

### Variable importance

Two methods for determining the most important predictors of revision risk were compared: backwards elimination in the Cox model and minimal depth from the random survival forest.

Random survival forest minimal depth uses the structure of survival trees in the forest to assess the variable importance by measuring the depth of each variable relative to the root node of the tree [20]. A small minimal depth indicates that the variable was chosen early in the splitting process, which implies it has a strong influence in determining the risk of revision for joint replacement. Minimal depth for each variable was averaged across 500 trees grown from a tuned random survival forest. Backwards elimination in the Cox model was performed with no stopping criterion and the order in which predictors were sequentially removed from the model was used to rank their importance. This process was repeated on 500 bootstrap samples of the data as variable selection from backward elimination is notoriously unstable [17]. Ranks were averaged across bootstrap samples and 95% confidence intervals for ranks were calculated assuming a normal distribution. This rank-based approach was used to allow more direct comparison to the minimal depth from the random survival forest. Backwards elimination was not performed for the parametric regression approaches, as model coefficients were nearly identical to the Cox model (as shown in Additional file 2).

Statistical analyses were performed using R version 3.6.3 (R Foundation for Statistical Computing, Vienna, Austria) with packages survival [33], bshazard [24], pec [34], riskRegression [35], flexsurv [36], rms [37], and randomForestSRC [38].

## Results

**Fig. 1:**
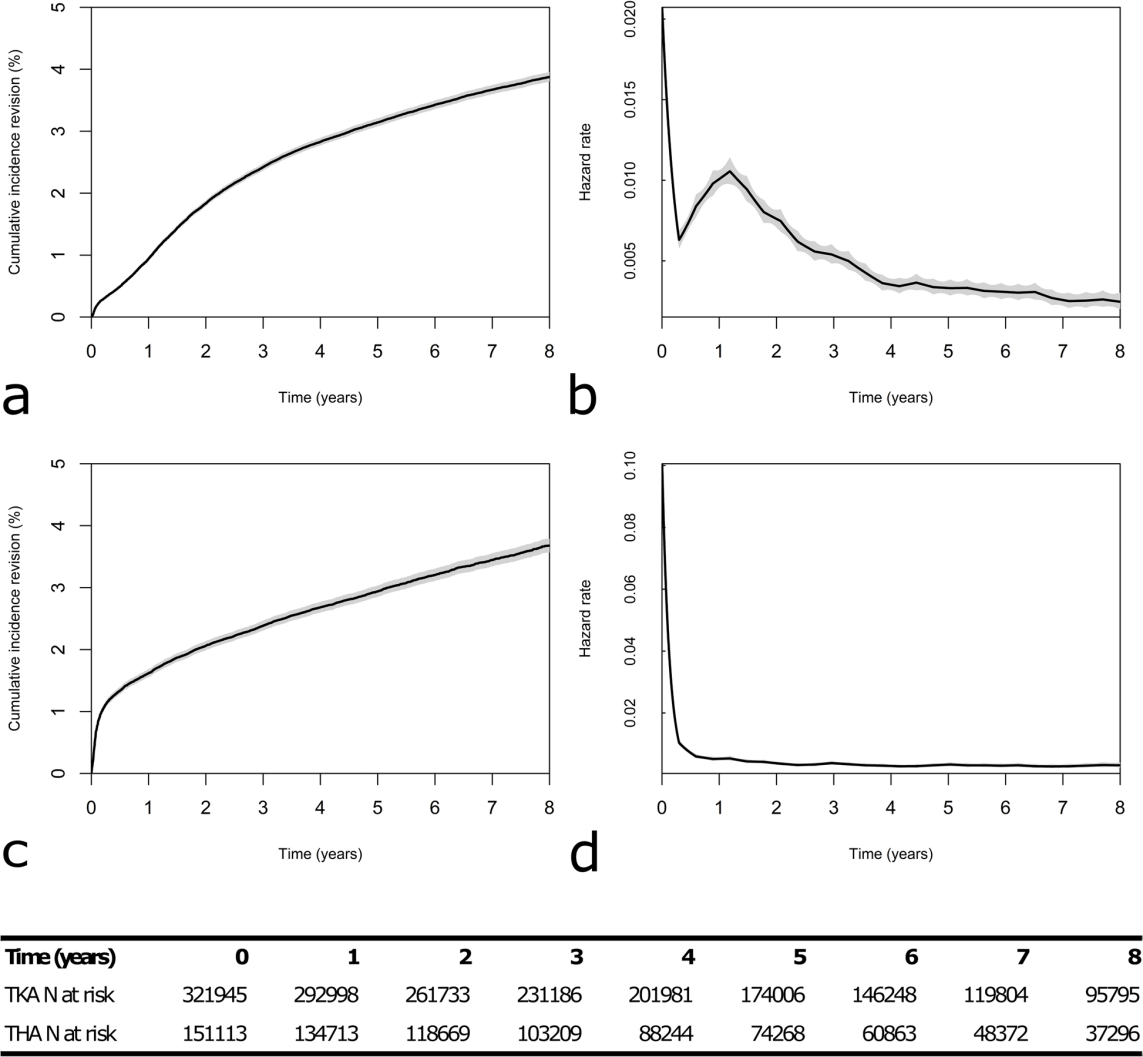
**a** Cumulative incidence of revision of TKA over 8 years. **b** Baseline hazard of TKA revision over 8 years. **c** Cumulative incidence of revision of THA over 8 years. **d** Baseline hazard of THA revision over 8 years. Shading indicates 95% confidence intervals

For TKAs, the cumulative incidence of revision was 3.9% at 8 years and the hazard function was non-monotonic; revision risk was highest initially after surgery, but spiked approximately 1 year after surgery before decreasing again (Fig. 1a and b). For THAs, the cumulative incidence of revision was 3.7% at 8 years and the hazard function monotonically decreased over time, with the risk of revision highest immediately after surgery (Fig. 1c and d). The median follow-up was 5.5 years for TKAs and 4.9 years for THAs. At 8 years 17% of TKA procedures and 19.6% of THA procedures were censored due to patient death.

For both TKA and THA, the discrimination of the four modelling approaches was virtually identical (c-index 0.64 for all four approaches for TKA revision and 0.59 for all four approaches for THA revision (Tables 3 and 4).

**Table 3:** Performance metrics for predicting revision of TKA using Cox, Weibull parametric, flexible parametric, and random survival forest models

TKA			
Modelling Approach	c-index	Integrated calibration index (× 100)	Integrated Index of Prediction Accuracy
Cox	0.643 (0.641, 0.645)	0.16 (0.15, 0.18)	0.056 (0.054, 0.057)
Weibull	0.642 (0.641, 0.644)	0.17 (0.16, 0.19)	0.054 (0.053, 0.056)
Flexible parametric	0.643 (0.641, 0.645)	0.17 (0.15, 0.18)	0.056 (0.054, 0.057)
Random survival forest	0.643 (0.642, 0.645)	0.27 (0.25, 0.29)	0.055 (0.054, 0.056)

**Table 4:** Performance metrics for predicting revision of THA using Cox, Weibull parametric, flexible parametric, and random survival forest models

THA			
	c-index	Integrated calibration index (×100)	Integrated Index of Prediction Accuracy
Cox	0.591 (0.589, 0.594)	0.27 (0.25, 0.3)	0.029 (0.027, 0.03)
Weibull	0.591 (0.588, 0.594)	0.29 (0.26, 0.31)	0.028 (0.026, 0.029)
Flexible parametric	0.591 (0.588, 0.594)	0.28 (0.25, 0.3)	0.029 (0.027, 0.030)
Random survival forest	0.59 (0.587, 0.592)	0.28 (0.25, 0.3)	﻿0.027 (0.025, 0.028)

**Fig. 2:**
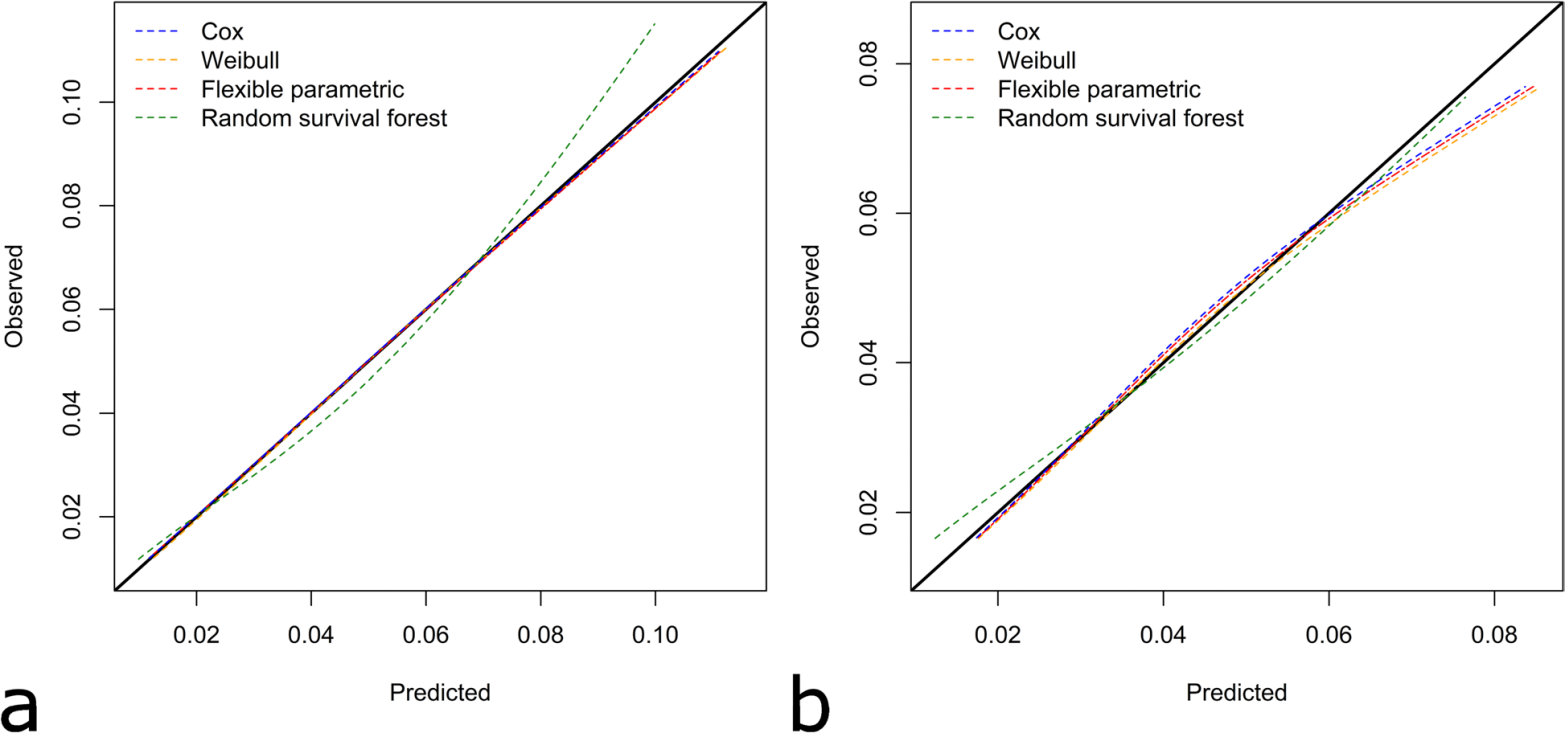
The calibration of models predicting eight-year risk of revision are compared using smoothed calibration curves, with black diagonal line denoting line of perfect calibration for **a** TKA and **b** THA

For TKAs the random survival forest had worse calibration than the Cox, Weibull or flexible parametric models. The ICI showed that on average, predicted risks from the Cox model differed from actual risk by 0.16% (95% CI [0.15, 0.18]), but this difference was 0.27% (95% CI [0.25,0.29]) for the random survival forest (Table 3). The Cox, Weibull, and flexible parametric models were well calibrated across the range of possible risks, whereas the random survival forest overestimated the risk for lower risk patients and underestimated the risk for higher risk patients (Fig. 2a).

For THA, all modelling approaches had similar overall calibration according to the ICI (Table 4). The Cox, Weibull and flexible parametric models were well calibrated for low-risk patients but overestimated the revision risk for higher risk patients. Conversely, the random survival forests were well calibrated in those with high risk but underestimated the risk for lower risk patients (Fig. 2b).

**Fig. 3:**
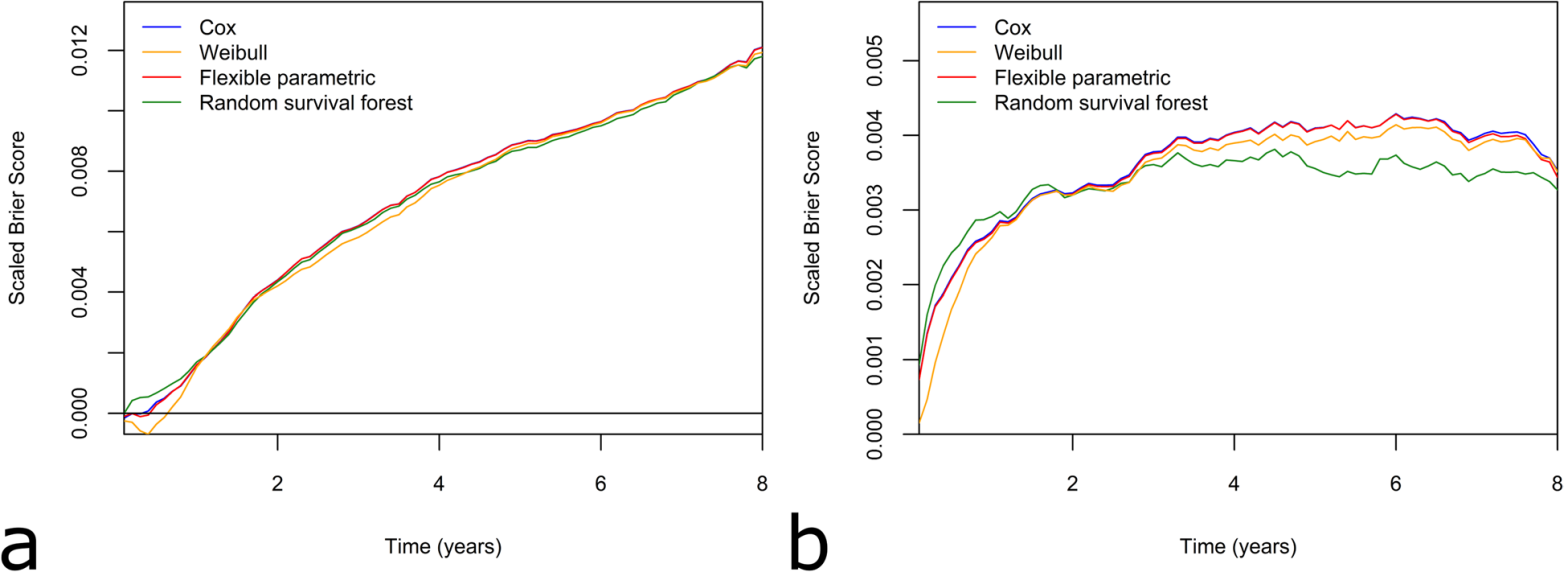
The Index of Prediction Accuracy is used to compare prediction accuracy of Cox, Weibull, flexible parametric and random survival forest for prediction of revision over eight-year time period for **a** TKA and **b** THA

When predicting TKA revision, the Cox and flexible parametric models returned the highest integrated IPA, each with a value of 0.056 (95% CI [0.054, 0.057]) while the Weibull model had the lowest IPA of 0.054 (95% CI [0.053, 0.056]) (Table 3). All models performed similarly in the later follow-up period, with the Weibull and random survival forest slightly worse (Fig. 3a). Within the first year of TKA, the random survival forest was the best performing approach for prediction of revision. In this earlier time period, the Weibull model had negative IPA, implying it performed worse than the null model.

When predicting THA revision, the Cox and flexible parametric models had the highest integrated IPA (0.029 95% CI [0.027, 0.030] compared to 0.027 (95% CI [0.025, 0.028]) for the random survival forest) (Table 4). The random survival forest had the highest IPA for revisions within the first 2 years but showed poorer performance than the other modelling approaches for later time periods. The Weibull model had slightly worse performance than the Cox and flexible parametric models over the entire eight-year period (Fig. 3b).

For TKA, rankings of variable importance from backwards elimination and random survival forest minimal depth identified the same three most important predictors of revision (age, use of pain medication (opioids), and use of patella resurfacing). Both selection methods ranked prosthesis stability, prosthesis bearing surface and patient depression as the three next most important predictors, with the order differing between methods (Table 5).

**Table 5:** Ranked importance of variables from backward elimination in Cox model compared to minimal depth from random survival forest, for prediction of TKA revision. Variables are displayed in decreasing order of importance

Bootstrap backwards elimination in Cox model (95% CI)		Minimal depth from random survival forest (95% CI)	
Age	1 (1,1)	Age	1.54 (1.45,1.64)
Pain	2.26 (2.21,2.3)	Pain	1.91 (1.8,2.02)
Patella usage	2.92 (2.86,2.97)	Patella usage	2.04 (1.93,2.15)
Stability	4.39 (4.34,4.44)	Depression	2.2 (2.08,2.32)
Bearing surface	4.51 (4.43,4.59)	Bearing surface	2.46 (2.34,2.58)
Depression	6.22 (6.17,6.28)	Stability	2.79 (2.68,2.9)
Sex	7.39 (7.32,7.46)	Mobility	3.05 (2.91,3.18)
Tibial cement	8.8 (8.62,8.98)	Anxiety	3.05 (2.91,3.19)
Anxiety	10.6 (10.44,10.77)	Sex	3.24 (3.13,3.35)
Ischaemic heart disease angina	10.68 (10.48,10.87)	Tibial cement	3.49 (3.36,3.62)
Mobility	10.77 (10.6,10.95)	Inflammation pain	4.2 (4.07,4.33)
Gastro-oesophageal reflux disease	11.02 (10.81,11.23)	Ischaemic heart disease angina	4.2 (4.09,4.32)
Steroid responsive diseases	12.48 (12.27,12.7)	Steroid responsive diseases	4.23 (4.11,4.35)
Computer navigation	16.48 (16.22,16.74)	Gastro-oesophageal reflux disease	4.28 (4.15,4.41)
congestive heart failure	16.89 (16.5,17.28)	Hypertension	4.47 (4.35,4.6)
Arrhythmia	17.1 (16.74,17.46)	Ischaemic heart disease hypertension	4.68 (4.56,4.8)
Ischaemic heart disease hypertension	18.62 (18.29,18.95)	Computer navigation	4.73 (4.62,4.83)
Hypothyroidism	19.16 (18.82,19.5)	congestive heart failure	4.74 (4.63,4.84)
Hypertension	19.39 (19.02,19.76)	Gout	4.74 (4.64,4.84)
Osteoporosis/Paget’s	19.88 (19.53,20.23)	Chronic Airways Disease	4.78 (4.66,4.9)
Anticoagulants	21.74 (21.37,22.12)	Femoral cement	4.86 (4.74,4.98)
Inflammation pain	22.09 (21.76,22.42)	Glaucoma	4.93 (4.81,5.05)
Chronic Airways Disease	22.96 (22.61,23.31)	Anticoagulants	4.93 (4.82,5.04)
Diabetes	23.9 (23.58,24.22)	Osteoporosis/Paget’s	5.1 (4.99,5.21)
Hyperlipidaemia	24.12 (23.82,24.41)	Arrhythmia	5.11 (5,5.21)
Antiplatelet medication	24.28 (23.96,24.6)	Diabetes	5.17 (5.07,5.27)
Gout	24.86 (24.59,25.14)	Antiplatelet medication	5.18 (5.07,5.29)
Glaucoma	25.23 (24.95,25.52)	Hypothyroidism	5.19 (5.1,5.29)
Femoral cement	25.24 (24.97,25.51)	Hyperlipidaemia	5.26 (5.16,5.36

For THA, rankings of variable importance from backwards elimination and random survival forest minimal depth identified the same five most important predictors of revision in the same order: femoral cement, patient depression, use of pain medication (opioids), gastro-oesophageal reflux disease, and sex. Patient age and steroid responsive diseases were the next most important predictors, with the ordering swapped between methods (Table 6).

**Table 6:** Ranked importance of variables from backward elimination in Cox model compared to minimal depth from random survival forest, for prediction of THA revision. Variables are displayed in decreasing order of importance

Bootstrap backwards elimination in Cox model (95% CI)		Minimal depth from random survival forest (95% CI)	
Femoral cement	1.46 (1.43,1.49)	Femoral cement	0.66 (0.62,0.7)
Depression	1.92 (1.87,1.98)	Depression	0.99 (0.93,1.04)
Pain	2.82 (2.76,2.89)	Pain	1.5 (1.44,1.55)
Gastro-oesophageal reflux disease	5.33 (5.19,5.46)	Gastro-oesophageal reflux disease	2.44 (2.36,2.51)
Sex	5.58 (5.49,5.67)	Sex	2.7 (2.65,2.76)
Age	7.49 (7.32,7.67)	Steroid responsive diseases	2.77 (2.69,2.85)
Steroid responsive diseases	8.29 (8.06,8.52)	Age	3.1 (3.05,3.15)
Inflammation pain	8.96 (8.79,9.13)	Congestive heart failure	3.25 (3.18,3.32)
Anxiety	9.74 (9.53,9.95)	Anxiety	3.64 (3.55,3.74)
Congestive heart failure	12.22 (11.93,12.5)	Bearing Surface	3.74 (3.67,3.81)
Osteoporosis/Paget’s	12.22 (11.98,12.46)	Head size	3.88 (3.83,3.94)
Head size	12.23 (11.97,12.49)	Osteoporosis/Paget’s	3.93 (3.84,4.02)
Bearing surface	12.32 (12.1,12.55)	Gout	4.18 (4.1,4.26)
Hyperlipidaemia	12.37 (12.19,12.54)	Inflammation pain	4.32 (4.23,4.41)
Anticoagulant	15.47 (15.18,15.76)	Acetabular cement	4.33 (4.26,4.41)
Hypothyroidism	16.74 (16.48,17)	Hyperlipidaemia	4.6 (4.51,4.69)
Chronic Airways Disease	17.61 (17.34,17.88)	Chronic Airways Disease	4.78 (4.69,4.87)
Gout	19.13 (18.86,19.4)	Anticoagulants	4.95 (4.85,5.04)
Arrhythmia	19.92 (19.64,20.2)	Arrhythmia	5.05 (4.95,5.15)
Ischaemic heart disease hypertension	20 (19.76,20.23)	Hypothyroidism	5.28 (5.19,5.38)
Antiplatelet medications	21.3 (21.09,21.52)	Ischaemic heart disease angina	5.32 (5.22,5.43)
Glaucoma	21.4 (21.2,21.6)	Hypertension	5.48 (5.38,5.58)
Diabetes	21.53 (21.33,21.74)	Ischaemic heart disease hypertension	5.55 (5.46,5.64)
Ischaemic heart disease angina	21.62 (21.42,21.82)	Antiplatelet medications	5.57 (5.47,5.67)
Acetabular cement	21.64 (21.45,21.84)	Diabetes	5.74 (5.64,5.85)
Hypertension	21.69 (21.48,21.89)	Glaucoma	6.11 (6,6.21)

## Discussion

This study found that the Cox and flexible parametric models outperformed the Weibull parametric model and random survival forest in the prediction of time-to-revision following either THA or TKA. Unsurprisingly, the flexible parametric model always outperformed the simpler Weibull model, particularly for TKA revision where the hazard function was complex and non-monotonic.

Random survival forests did not outperform carefully constructed regression models, despite being optimised in a large training set. This result is consistent with the findings of a systematic review that found no evidence machine learning provides improved performance over logistic regression in the binary outcome setting [39]. However, a review has not yet been conducted for time-to-event outcomes, where machine learning approaches have the additional advantage of not being constrained by the proportional hazards assumption.

A recent review highlighted the need for more studies comparing the prediction accuracy of the Royston-Parmar flexible parametric model to that of the Cox model [18]. Our study demonstrated that the flexible parametric approach had near-identical prediction accuracy to the Cox model. However, our results are in contrast to those of Aram et al., who found the flexible parametric model outperformed both Cox regression and random survival forests for prediction of eight-year revision of TKA [40], albeit with fewer predictors considered than the present study.

Ultimately, the overall performance indicated by the Brier score was very similar across the four modelling approaches, despite their different underlying model assumptions. The random survival forest is not constrained by the proportional hazards assumption and is also able to automatically model variable interactions, so its failure to outperform regression models here may indicate that time-varying effects of variables and variable interactions are not strongly predictive of revision surgery. The rarity of the predicted outcome and low signal-noise ratio may also contribute to the similarity of prediction performance in this setting.

Our results identified that ranking of important predictors was similar when using backwards elimination in the Cox model and minimal depth in random survival forests, suggesting that the important predictors identified are relatively robust to selection method. Many of the variables important in predicting revision risk were prosthesis-related, rather than patient-related. This was particularly true for TKAs, where patella resurfacing, prosthesis stability and bearing surface were among the six most important revision factors. However, for THA, use of femoral cement and several comorbid conditions were identified as important risk factors of revision. The presence of pain, identified by opioid usage, was predictive of revision risk in both THA and TKA patients, consistent with the association between pre-operative opioid use and increased revision risk documented in other studies [41–44]. Depression was also identified as an important risk factor in both TKA and THA revision. In a study of the effect of 26 comorbidities on revision rates, depression was found to have the strongest effect on revision risk [45]. Gastro-oesophageal reflux disease was identified as an important predictor of THA revision, possibly reflecting the association between the use of proton pump inhibitors and increased risk of hip fracture [46, 47].

A limitation of this study was the relatively low prediction accuracy (c-index of 0.64 for TKA and 0.59 for THA), which may have been due to the absence of certain patient and surgical factors from the dataset, such as patient body mass index, frailty, socioeconomic measures, lifestyle factors, and comorbidities not treated with indicative prescription medication. However, the models reported here performed similarly to other prediction models for TKA revision developed using registry data [40, 48], and all models were shown to outperform the null model with no covariates (IPA value > 0), indicating there is value in developing a predictive model in this setting.

Competing risks were not considered in this study, as a previous study in this setting has shown negligible difference in prediction accuracy between Cox regression and competing risk alternatives [21]. However, the comparative performance of competing risks extensions to the flexible parametric and random survival forest models could be explored in future research. This study also did not consider interaction terms nor time-varying coefficients in the regression models. Flexible parametric survival models can easily incorporate time-varying effects in auxiliary parameters and could be explored in future research. However, given that we did not see improved performance from the random survival forest, which automatically models interactions and time varying effects, this may indicate that limited performance gain will be realized by modelling interactions and time-varying effects in this setting. Future work could also compare the performance of other machine learning approaches available for time-to-event data, including support vector machines [49], neural nets [50–52], and gradient boosting [53, 54].

## Conclusion

The Cox and flexible parametric models were shown to have superior accuracy for predicting time-to-revision risk following TKA and THA compared to random survival forests. The Cox model and random survival forest also identified similar predictors as being the most important for revision risk. Our findings suggest that random survival forests for risk prediction models in the joint replacement setting offer no benefit over regression approaches in terms of prediction accuracy and give broadly similar conclusions regarding variable importance.

## Supplementary Information

**Additional file 1.** Flowcharts showing inclusion criteria for TKA and THA procedures used in the analysis. This file contains flowcharts showing detailed inclusion criteria and number of procedures excluded as a result for TKA and THA.

**Additional file 2.** Hazard ratios and plots of fitted restricted cubic spline for age for TKA and THA. This file contains tables showing the hazard ratios from Cox, Weibull and flexible parametric models fitted to the full dataset for TKA and THA revisions, as well as plots of the restricted cubic splines fit for age (as these difficult to summarise numerically).

**Additional file 3.** Details of random survival forest tuning. This file details the parameters and process used to tune random survival forests presented in the manuscript.

**Additional file 4.** Plots of overall calibration. This file contains additional calibration plots comparing Kaplan-Meier estimates of survivorship to the average of predicted survival curves for each of the four methods.

## Data Availability

The data for this study was creating from pre-existing datasets (Australian Orthopaedic Association National Joint Replacement Registry (AOANJRR) and routinely-collected Australian Government Medicare Benefits Schedule and Pharmaceutical Benefits Scheme data), with data custodian permissions and specific ethical approval. The data may potentially be made available to other researchers if they obtain the necessary approvals. Further information on the process is available from the following sources: Population Health and Research Network (https://www.phrn.org.au/for-researchers/data-access/cross-and-multi-jurisdictional-application-process/), AOANJRR (https://aoanjrr.sahmri.com/aoanjrr-data-linkage, admin@aoanjrr.org.au) and the Australian Institute of Health and Welfare (https://www.aihw.gov.au/our-services/data-linkage, linkage@aihw.gov.au).

## Abbreviations

THA: Total Hip Arthroplasty
TKA: Total Knee Arthroplasty
AOANJRR: Australian Orthopaedic Association National Joint Replacement Registry
CI: Confidence interval
IPA: Index of prediction accuracy
ICI: Integrated calibration index
