# Supplementary material for: A comparison of survival models for prediction of eight-year revision risk following total knee and hip arthroplasty
Source: BMC Med Res Methodol. 2022 Jun 6;22:164. doi: 10.1186/s12874-022-01644-3 (PMC9172144; doi:10.1186/s12874-022-01644-3)

Supplementary Figure 1: Flowchart showing inclusion criteria for primary TKA procedures included in the study.

**
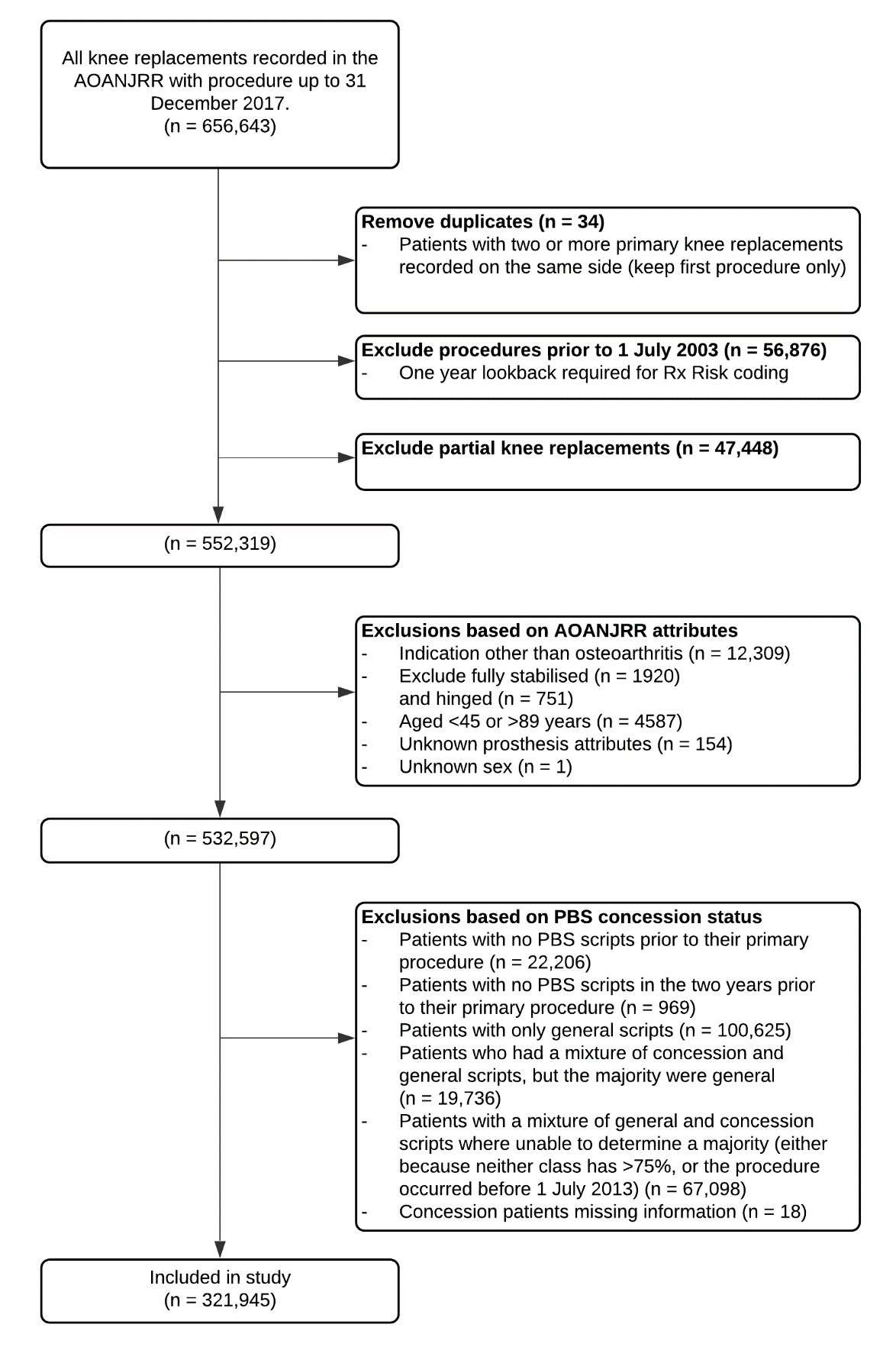
**

Supplementary Figure 2: Flowchart showing inclusion criteria for primary THA procedures included in the study.


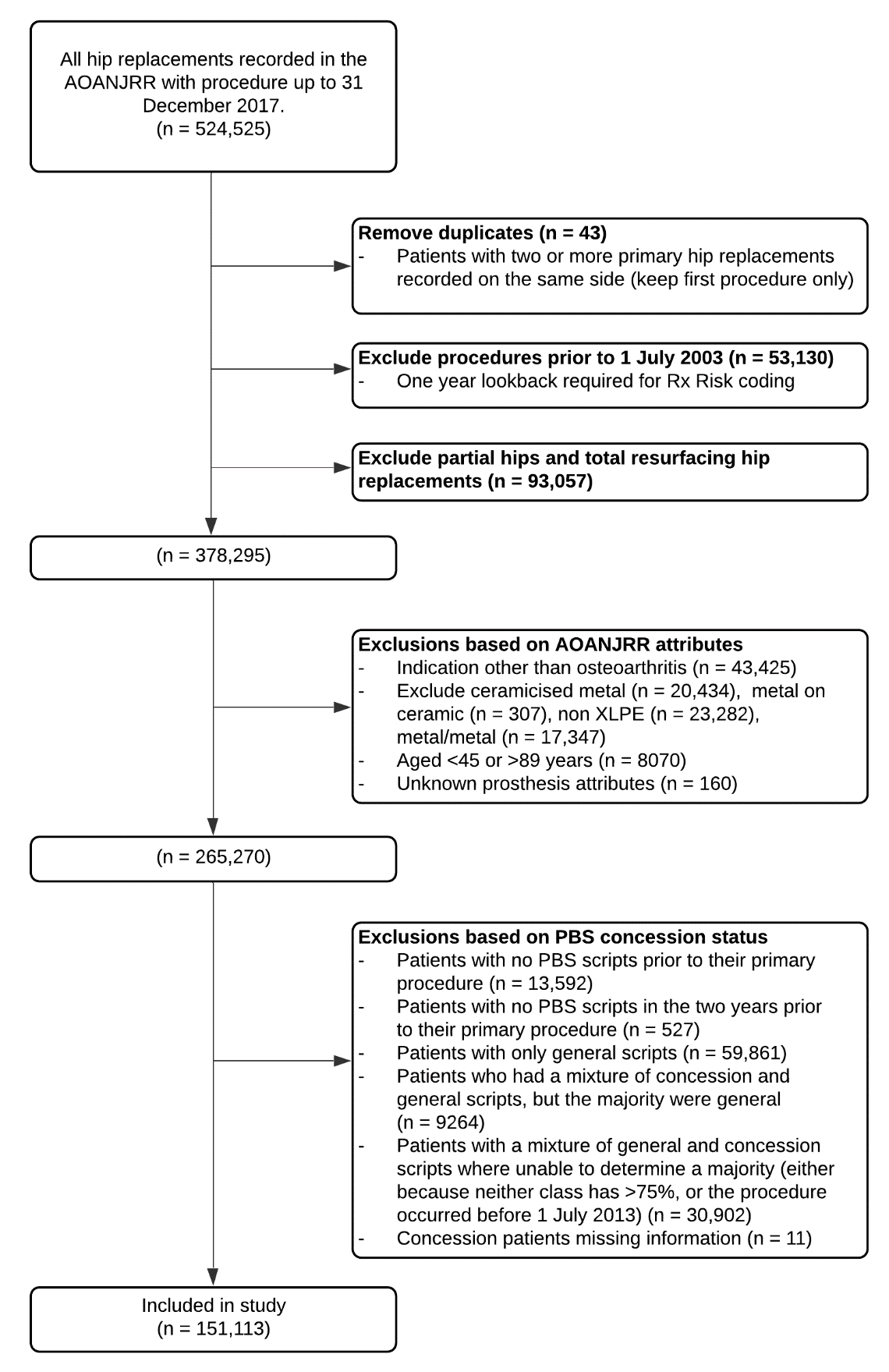

Supplement: Supplementary file 1 — Additional file 1. Flowcharts showing inclusion criteria for TKA and THA procedures used in the analysis. This file contains flowcharts showing detailed inclusion criteria and number of procedures excluded as a result for TKA and THA. [file 12874_2022_1644_MOESM1_ESM.docx]
