# Supplementary material for: A comparison of survival models for prediction of eight-year revision risk following total knee and hip arthroplasty
Source: BMC Med Res Methodol. 2022 Jun 6;22:164. doi: 10.1186/s12874-022-01644-3 (PMC9172144; doi:10.1186/s12874-022-01644-3)

Supplementary Table 1: Hazard Ratios from Cox, Weibull and flexible parametric models with 95% confidence intervals for revision for TKA

| **Variable** | **Cox** | **Weibull** | **Flexible Parametric** |
| --- | --- | --- | --- |
| rcs(age, 4)age | 0.96 (0.96, 0.97) | 0.96 (0.96, 0.97) | 0.96 (0.96, 0.97) |
| rcs(age, 4)age' | 0.99 (0.98, 1.01) | 0.99 (0.98, 1.01) | 1 (0.98, 1.02) |
| rcs(age, 4)age'' | 1 (0.9, 1.11) | 1 (0.91, 1.11) | 0.95 (0.85, 1.05) |
| Male vs female | 1.21 (1.17, 1.27) | 1.22 (1.17, 1.28) | 1.21 (1.16, 1.26) |
| Patella used vs no patella | 0.7 (0.68, 0.73) | 0.71 (0.68, 0.74) | 0.7 (0.67, 0.73) |
| Non-navigated vs navigated | 0.92 (0.87, 0.97) | 0.9 (0.85, 0.95) | 0.92 (0.87, 0.97) |
| Posterior vs minimally stabilised | 1.38 (1.32, 1.45) | 1.38 (1.32, 1.45) | 1.38 (1.32, 1.45) |
| Non XLPE vs XLPE | 1.33 (1.26, 1.4) | 1.27 (1.21, 1.34) | 1.33 (1.26, 1.4) |
| Mobile vs fixed bearing | 1.14 (1.09, 1.2) | 1.14 (1.09, 1.19) | 1.15 (1.09, 1.2) |
| Cementless vs cemented femoral | 1 (0.95, 1.05) | 0.99 (0.94, 1.04) | 1 (0.95, 1.05) |
| Cementless vs cemented tibial | 1.17 (1.1, 1.23) | 1.17 (1.1, 1.23) | 1.17 (1.11, 1.24) |
| Anticoagulants | 1.04 (0.98, 1.11) | 1.05 (0.99, 1.12) | 1.05 (0.99, 1.12) |
| Antiplatelet medications | 1.02 (0.97, 1.07) | 1.01 (0.96, 1.06) | 1 (0.95, 1.06) |
| Anxiety | 1.17 (1.11, 1.24) | 1.17 (1.1, 1.24) | 1.17 (1.1, 1.23) |
| Arrhythmia | 1.12 (1.02, 1.22) | 1.12 (1.02, 1.22) | 1.12 (1.02, 1.22) |
| Congestive Heart Failure | 1.1 (1.02, 1.18) | 1.1 (1.02, 1.18) | 1.09 (1.01, 1.17) |
| Depression | 1.2 (1.15, 1.25) | 1.21 (1.16, 1.26) | 1.2 (1.14, 1.25) |
| Diabetes | 0.98 (0.92, 1.03) | 0.98 (0.93, 1.04) | 0.97 (0.92, 1.03) |
| Gastro-oesophageal Reflux Disease | 1.1 (1.06, 1.15) | 1.11 (1.06, 1.15) | 1.11 (1.07, 1.16) |
| Glaucoma | 1 (0.92, 1.09) | 1 (0.92, 1.09) | 1.03 (0.94, 1.12) |
| Gout | 1 (0.94, 1.07) | 1 (0.93, 1.07) | 1.01 (0.95, 1.08) |
| Hyperlipidaemia | 1.02 (0.97, 1.06) | 1.03 (0.98, 1.07) | 1.02 (0.98, 1.07) |
| Hypertension | 0.96 (0.92, 1) | 0.96 (0.92, 1) | 0.94 (0.91, 0.98) |
| Hypothyroidism | 1.07 (1, 1.14) | 1.08 (1.01, 1.15) | 1.07 (1, 1.14) |
| Ischaemic heart disease (angina) | 1.2 (1.12, 1.3) | 1.19 (1.11, 1.29) | 1.18 (1.09, 1.27) |
| Ischaemic heart disease (hypertension) | 0.95 (0.91, 0.99) | 0.95 (0.91, 0.99) | 0.94 (0.9, 0.98) |
| Osteoporosis/Paget’s | 1.07 (1, 1.15) | 1.07 (1, 1.15) | 1.06 (0.99, 1.14) |
| Pain | 1.3 (1.25, 1.36) | 1.31 (1.26, 1.37) | 1.3 (1.25, 1.35) |
| Inflammation pain | 1.03 (0.99, 1.07) | 1.01 (0.97, 1.05) | 1.02 (0.98, 1.06) |
| Chronic airways disease | 1.03 (0.98, 1.08) | 1.03 (0.99, 1.08) | 1.02 (0.97, 1.07) |
| Steroid responsive | 1.11 (1.06, 1.17) | 1.11 (1.06, 1.17) | 1.12 (1.06, 1.18) |

Supplementary Figure 3: Restricted cubic spline estimate of relationship between age and relative log hazard of TKA revision. The Wald statistic for test of non-linearity is χ² = 3.35, P = 0.188.


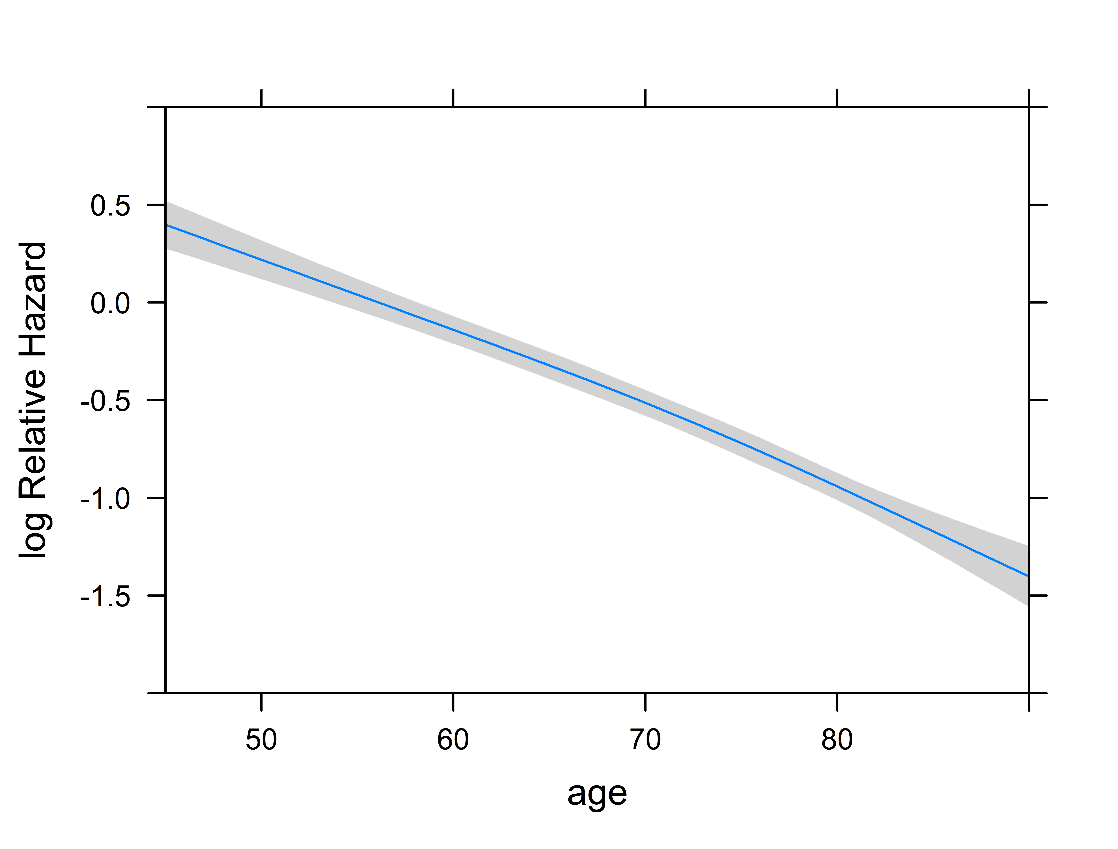


Supplementary Table 2: Hazard ratios and 95% confidence intervals from Cox, Weibull and flexible parametric models for THA revision

| Variable | Cox | Weibull | Flexible parametric |
| --- | --- | --- | --- |
| rcs(age, 4)age | 0.98 (0.98, 0.99) | 0.98 (0.98, 0.99) | 0.98 (0.98, 0.99) |
| rcs(age, 4)age' | 1.05 (1.02, 1.07) | 1.05 (1.02, 1.07) | 1.05 (1.02, 1.07) |
| rcs(age, 4)age'' | 0.83 (0.71, 0.96) | 0.83 (0.71, 0.96) | 0.83 (0.71, 0.96) |
| Male sex | 1.22 (1.14, 1.3) | 1.22 (1.15, 1.3) | 1.22 (1.14, 1.3) |
| Ceramic/XLPE vs ceramic/ceramic | 0.86 (0.78, 0.95) | 0.85 (0.77, 0.94) | 0.86 (0.78, 0.95) |
| Metal/XLPE vs ceramic/ceramic | 0.89 (0.82, 0.96) | 0.88 (0.81, 0.95) | 0.89 (0.82, 0.96) |
| Head size 32mm vs ≤28mm | 0.87 (0.81, 0.94) | 0.84 (0.78, 0.91) | 0.87 (0.81, 0.94) |
| Head size 36mm vs ≤28mm | 0.92 (0.84, 1) | 0.87 (0.8, 0.95) | 0.91 (0.84, 0.99) |
| Head size ≥40mm vs ≤28mm | 0.85 (0.69, 1.05) | 0.79 (0.64, 0.97) | 0.85 (0.69, 1.04) |
| Cementless vs cemented femoral | 1.49 (1.4, 1.59) | 1.49 (1.4, 1.6) | 1.49 (1.4, 1.59) |
| Cementless vs cemented acetabular | 0.97 (0.8, 1.19) | 0.99 (0.81, 1.21) | 0.97 (0.8, 1.19) |
| Anticoagulants | 1.09 (1, 1.19) | 1.09 (1, 1.19) | 1.09 (1, 1.19) |
| Antiplatelet medications | 1.02 (0.94, 1.1) | 1.02 (0.94, 1.1) | 1.02 (0.94, 1.1) |
| Anxiety | 1.18 (1.08, 1.28) | 1.18 (1.08, 1.28) | 1.18 (1.08, 1.28) |
| Arrhythmia | 1.05 (0.93, 1.19) | 1.05 (0.93, 1.19) | 1.05 (0.93, 1.19) |
| Congestive Heart Failure | 1.15 (1.04, 1.28) | 1.15 (1.03, 1.28) | 1.15 (1.04, 1.28) |
| depression | 1.34 (1.25, 1.43) | 1.33 (1.25, 1.42) | 1.34 (1.25, 1.43) |
| diabetes | 1.01 (0.92, 1.11) | 1 (0.91, 1.1) | 1.01 (0.92, 1.11) |
| Gastro-oesophageal Reflux Disease | 1.17 (1.1, 1.24) | 1.17 (1.1, 1.24) | 1.17 (1.1, 1.24) |
| glaucoma | 0.98 (0.87, 1.1) | 0.98 (0.87, 1.1) | 0.98 (0.87, 1.1) |
| gout | 1.07 (0.96, 1.18) | 1.06 (0.96, 1.18) | 1.07 (0.96, 1.18) |
| Hyperlipidaemia | 0.9 (0.85, 0.96) | 0.9 (0.84, 0.96) | 0.9 (0.85, 0.96) |
| Hypertension | 0.99 (0.93, 1.05) | 0.99 (0.93, 1.05) | 0.99 (0.93, 1.05) |
| Hypothyroidism | 1.1 (0.99, 1.21) | 1.09 (0.99, 1.21) | 1.1 (0.99, 1.21) |
| Ischaemic heart disease (angina) | 1.01 (0.9, 1.13) | 1.01 (0.9, 1.14) | 1.01 (0.9, 1.13) |
| Ischaemic heart disease (hypertension) | 1.03 (0.97, 1.1) | 1.03 (0.97, 1.1) | 1.03 (0.97, 1.1) |
| Osteoporosis/Paget’s | 1.16 (1.05, 1.27) | 1.16 (1.05, 1.27) | 1.16 (1.05, 1.27) |
| Pain | 1.23 (1.15, 1.3) | 1.22 (1.15, 1.3) | 1.23 (1.15, 1.3) |
| Inflammation pain | 1.14 (1.08, 1.22) | 1.16 (1.09, 1.23) | 1.15 (1.08, 1.22) |
| Chronic airways disease | 1.06 (0.99, 1.14) | 1.06 (0.98, 1.14) | 1.06 (0.99, 1.14) |
| Steroid responsive | 1.16 (1.07, 1.25) | 1.16 (1.07, 1.25) | 1.16 (1.07, 1.25 |

Supplementary Figure 4: Restricted cubic spline estimate of relationship between age and relative log hazard of THA revision. The Wald statistic for test of non-linearity is: χ² = 19.26, P<0.001


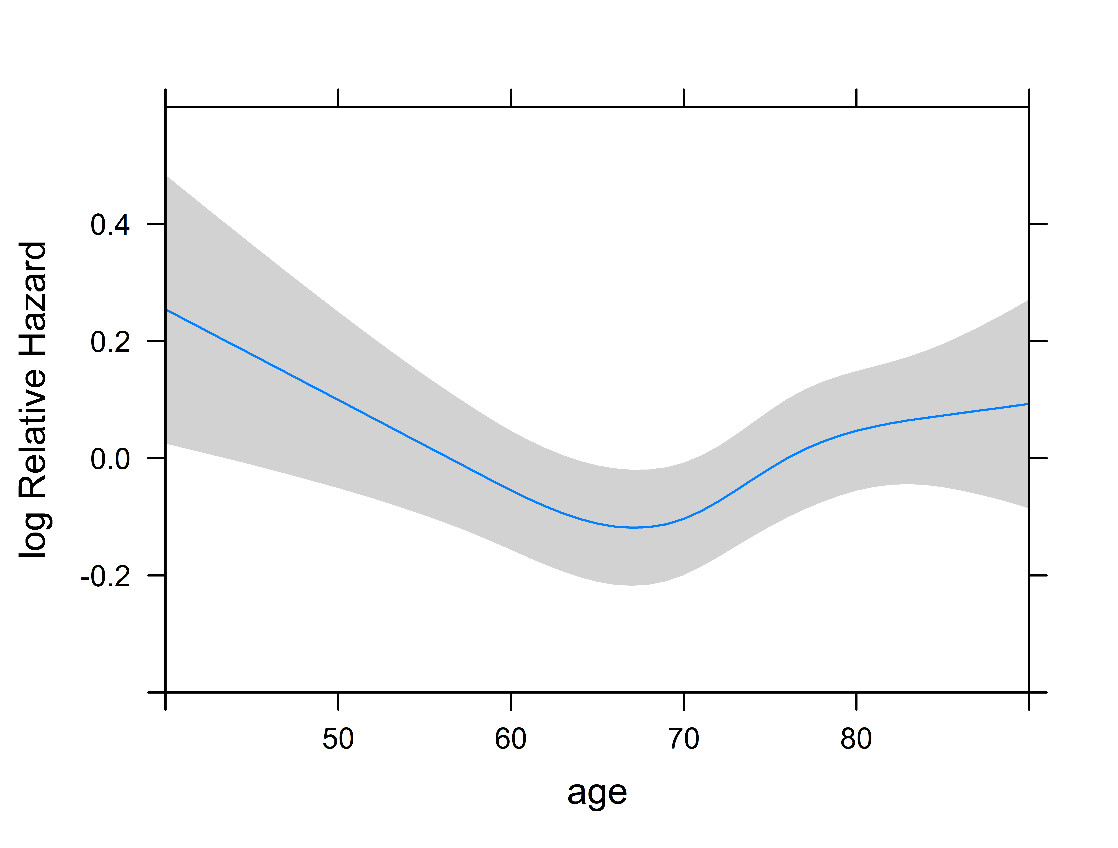

Supplement: Supplementary file 2 — Additional file 2. Hazard ratios and plots of fitted restricted cubic spline for age for TKA and THA. This file contains tables showing the hazard ratios from Cox, Weibull and flexible parametric models fitted to the full dataset for TKA and THA revisions, as well as plots of the restricted cubic splines fit for age (as these difficult to summarise numerically). [file 12874_2022_1644_MOESM2_ESM.docx]
