# Supplementary material for: A comparison of survival models for prediction of eight-year revision risk following total knee and hip arthroplasty
Source: BMC Med Res Methodol. 2022 Jun 6;22:164. doi: 10.1186/s12874-022-01644-3 (PMC9172144; doi:10.1186/s12874-022-01644-3)

**Details of tuning random survival forest:**

Two parameters for the random survival forest were tuned within each fold of cross- validation: *mtry* and *nodesize*. Within each fold, *ntree* = 200 trees were grown when tuning. This number was chosen because after examining the effect of adding more trees to the forest, there was minimal gain in prediction accuracy beyond 200 trees. An initial grid search was performed using the entire dataset in order to narrow down the number of parameter values considered during cross-validation and considerably reduce computing time.

Initial tuning was performed by identifying parameter values that minimised the out- of-bag (OOB) error from random survival forests grown using *ntree* = 100 trees in the entire dataset using rfsrc.fast (inbuilt function of randomForestSRC package that performs a fast approximation of the random survival forest using subsampling). To reduce computing time, *ntime* was restricted to 17 discrete time points: (0.1, 0.2, 0.3, 0.4, 0.5, 0.6, 0.7, 0.8, 0.9, 1, 2, 3, 4, 5, 6, 7, 8). *nsplit* was set to 5, again to reduce computing time, as there were only 49 distinct values of the continuous predictor age.

*TKA*

For TKA revision, a grid search across eight values of *nodesize* (15, 50, 100, 200, 350, 500, 750, 1000) and five values of *mtry* (3, 6, 10, 15, 20) was performed, for a total of 40 parameter value combinations. The minimum OOB error appeared to occur with a *nodesize* of 100-400 and *mtry* of 6 (Figure 1). Based on these results, during 10-fold cross-validation random survival forests were tuned using *mtry* = 6 and considered four values of *nodesize* (15, 100, 200, and 400) (15 was included as it is the default value in randomForestSRC).

*THA*

For THA revision, a grid search across 12 values of *nodesize* (15, 50, 100, 200, 250, 500, 750, 1000, 1250, 1500, 1750, 2000) and five values of *mtry* (3, 6, 10, 15, 20) was performed, for a total of 60 parameter value combinations. The minimum OOB error appeared to occur with a *nodesize* of 350-650 and *mtry* of 10-15 (Figure 4.8). Based on these results, during 10-fold cross-validation random survival forests were tuned by considering two values of *mtry* (10, 15) and four values of *nodesize* (15, 350, 500, 650)

The parameter values that resulted in the minimum OOB error in these initial grid searches were used when growing the random survival forests used to derive variable importance rankings via minimal depth with *ntree* = 500 trees. That is, *mtry* = 6 and *nodesize* = 200 for TKA, and *mtry* = 15 and *nodesize* = 500 for THA.

Supplementary Figure 5: Out of box error (1−c-index) from random survival forest for different values of *mtry* and *nodesize* for prediction of TKA revision


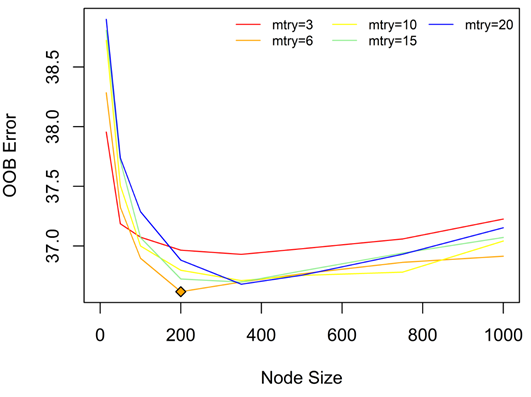


Supplementary Figure 6: Out of box error (1−c-index) from random survival forest for different values of *mtry* and *nodesize* for prediction of THA revision


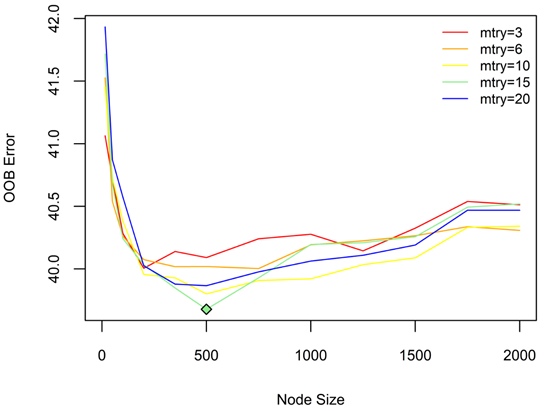

Supplement: Supplementary file 3 — Additional file 3. Details of random survival forest tuning. This file details the parameters and process used to tune random survival forests presented in the manuscript. [file 12874_2022_1644_MOESM3_ESM.docx]
