# Supplementary material for: A comparison of survival models for prediction of eight-year revision risk following total knee and hip arthroplasty
Source: BMC Med Res Methodol. 2022 Jun 6;22:164. doi: 10.1186/s12874-022-01644-3 (PMC9172144; doi:10.1186/s12874-022-01644-3)

**Plots of overall calibration**

Supplementary Figure 7: Kaplan-Meier estimates of survivorship compared to average of predicted survival curves for TKA revision for each method


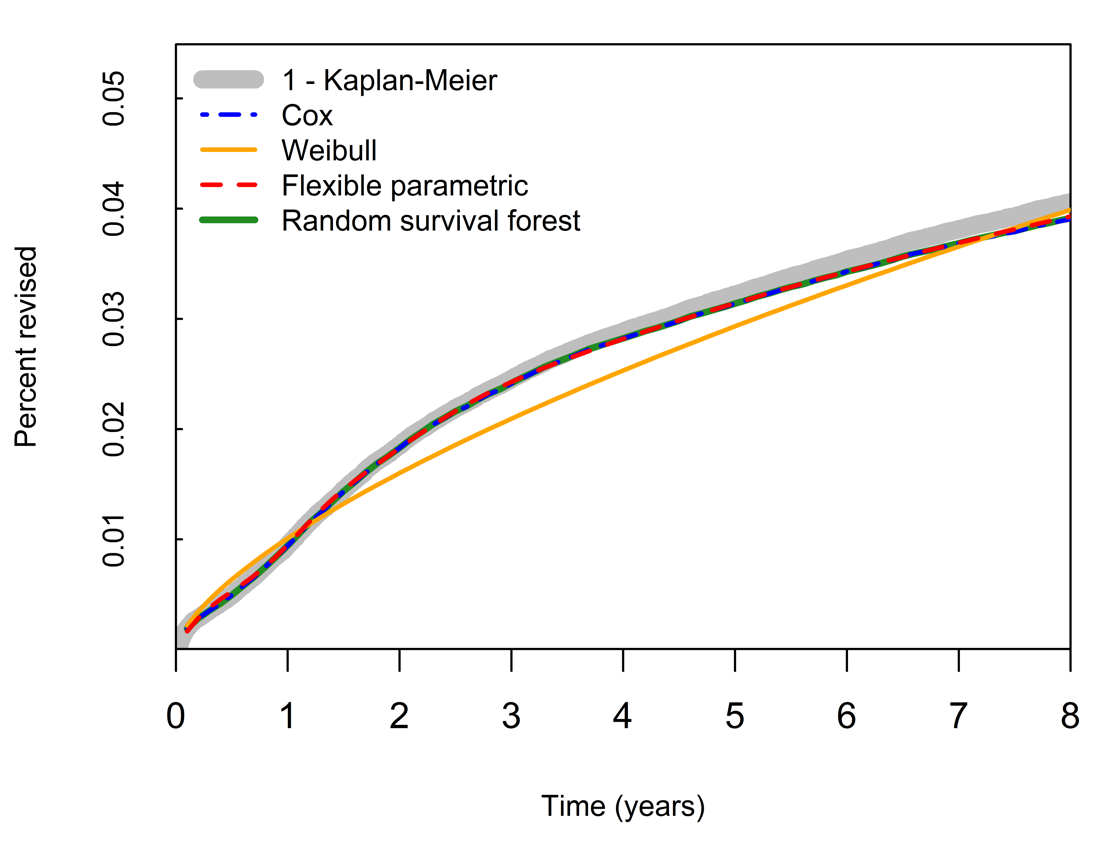


Supplementary Figure 8: Kaplan-Meier estimates of survivorship compared to average of predicted survival curves for THA revision for each method


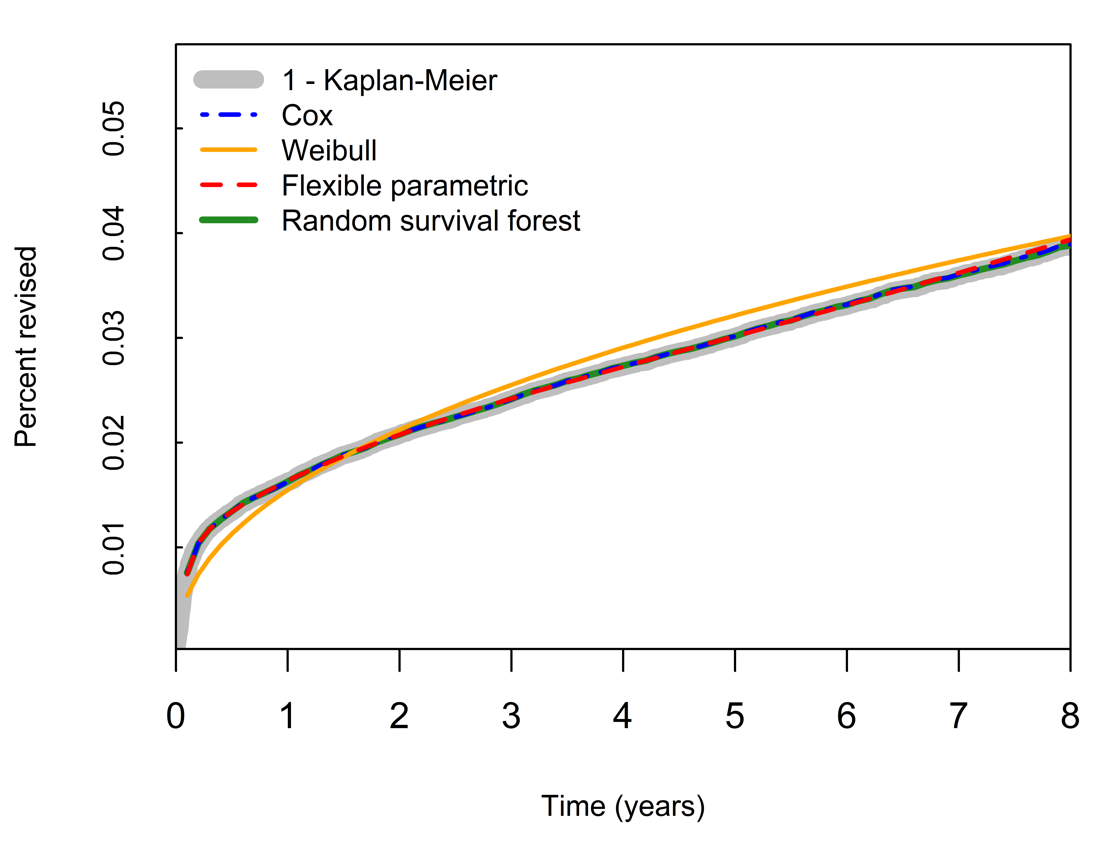

Supplement: Supplementary file 4 — Additional file 4. Plots of overall calibration. This file contains additional calibration plots comparing Kaplan-Meier estimates of survivorship to the average of predicted survival curves for each of the four methods. [file 12874_2022_1644_MOESM4_ESM.docx]
